# Food-Grade Bigel Systems: Formulation, Characterization, and Applications for Novel Food Product Development

**DOI:** 10.3390/gels10110712

**Published:** 2024-11-03

**Authors:** Konstantina Zampouni, Dafni Dimakopoulou-Papazoglou, Eugenios Katsanidis

**Affiliations:** Department of Food Science and Technology, School of Agriculture, Faculty of Agriculture, Forestry and Natural Environment, Aristotle University of Thessaloniki, 54124 Thessaloniki, Greece; ddimakop@agro.auth.gr (D.D.-P.); ekatsani@agro.auth.gr (E.K.)

**Keywords:** bigels, biphasic systems, oleogels, hydrogels, food applications

## Abstract

Bigels are characterized as biphasic systems consisting of two structured phases of different polarity, namely the oleogel and hydrogel phases. These systems have been widely used in pharmaceuticals and cosmetics, owing to their enhanced physicochemical stability compared to other structured systems and their ability to simultaneously deliver both hydrophilic and lipophilic compounds. Considering the above advantages, bigels could have considerable potential for the food industry. This review aims to provide a detailed description of the edible components used for structuring the oleogel and hydrogel phases and the preparation methods applied for the formation of food-grade bigels with application-specific tailored properties. The impact of the processing parameters, such as the oleogel-to-hydrogel ratio, methodology used for component mixing, and cooling rates, is presented. Moreover, the most applicable bigel characterization techniques, such as rheology, DSC, texture analysis, etc., are critically discussed. Finally, different bigel applications in foods as animal fat substitutes or as complex delivery systems for both polar and non-polar bioactive compounds are examined.

## 1. Introduction

The term “bigels” or “hybrid gels” was first suggested in 2008 by Almeida and coworkers [1] to explicate the combination of two discrete structured systems, an oleogel and a hydrogel, that are differentiated by their opposing polarity [2]. In most instances, the addition of emulsifiers is not required, as the mixing of the two phases results in the formation of a stable gel. Given that oleogels and hydrogels are recognized as effective systems for the delivery and release of bioactive compounds, it is evident that bigels possess the potential to transport both lipophilic and hydrophilic components individually and simultaneously [3]. The greater permeability of lipophilic bioactive substances through the skin when using bigel systems as carriers, compared to simple emulsions, oleogels, and hydrogels, is a reason for the extensive recent application of bigels in the field of pharmaceuticals and cosmetics [4,5].

The main advantage of bigels is their improved stability compared to emulsions, emulsion gels, hydrogels, and oleogels. This enhanced physicochemical stability is explained by the colloidal dispersion of one phase within the other, which renders these systems highly suitable and ideal for utilization in various food products [1,6]. Additionally, bigel systems typically do not exhibit any phase separation throughout storage at ambient temperatures, providing improved stability when incorporated into shelf-stable foods [7]. However, bigel stability can be influenced by several factors, such as the composition of the bigel, storage conditions, and interactions with food ingredients. Conversely, emulsion systems suffer from destabilization and phase separation over time. Thus, it is considered necessary to add an emulsifier to achieve the stabilization of an emulsion, in contrast to bigels [8]. However, it should be noted that, despite these advantages, bigels tend to destabilize when exposed to high temperatures; thereby, generally, they are not classified as thermoreversible systems [9].

Recently, the application of bigels in the field of food science has gained significant attention due to their potential use as fat-replacement systems. The benefit of the formation of a bigel-based fat-mimetic system is the ability to tailor its mechanical and functional properties. The utilization of bigels, which consist partly of a low-calorie hydrogel, can result in a reduced overall fat content compared to traditional solid fats. Moreover, bigels present an alternative solution for the development of nutritionally enriched foods. The biphasic nature of bigels allows the transport of various bioactive substances (e.g., lipids, pigments, vitamins), while their distinct physicochemical properties facilitate the controlled release of these components, providing them with additional protection [10,11,12]. Modulating the physicochemical and structural characteristics of bigels is very important to their successful incorporation into diverse food matrices [13].

This review focuses on recent advances in the development of the two structured phases (oleogels and hydrogels) of bigels and on the techniques for the successful preparation of a functional bigel system. Also, considering the importance of bigels for the food industry, an overview of the most used characterization methods for the assessment of the functionality of these systems is presented. Finally, the utilization of bigels in several food applications is also summarized.

## 2. Bigel Composition and Preparation Methods

### 2.1. Oleogels Used for Bigel Preparation

Over the past few years, oleogelation has increasingly attracted the interest of the food industry as an alternative to partial hydrogenation for the preparation of gels that successfully mimic hard fats. Oleogelation is a natural process which converts liquid edible oils into semi-solid gels, without modifying the chemical characteristics of the used oils [14]. The supramolecular organization of low- or high-molecular-weight oleogelators results in the formation of a thermoreversible three-dimensional (3D) network which restricts the mobility of hydrophobic liquid edible oils [15].

Various molecular interactions are responsible for the formation of oleogels, such as hydrogen bonds, π-π stacking, electrostatic interactions, and van der Waals interactions [16]. Proteins, polysaccharides, fatty acids, phytosterols, etc., can be used as oleogelators, depending on the desired physical properties of the food systems into which the obtained oleogels are to be incorporated. Process variables, such as the temperature, time, and mixing rate, play a crucial role in customizing the mechanical properties of the final oleogel. Also, the type and concentration of the oleogelator, the type of oil, and the proportions of the different structurants can modify the microstructure of oleogels, thereby impacting their textural parameters. Different methodologies can be applied to structure oil and form oleogels. The most frequently used method is direct dispersion, where lipid-soluble oleogelators are directly dissolved in the oil at elevated temperatures. Upon cooling, low-molecular-weight oleogelators self-assemble to form a 3D network which efficiently entraps the oil, resulting in an oleogel structure [17,18]. Nevertheless, to broaden the scope of applicable oleogelators like hydrophobic proteins and other polymers (except ethylcellulose), alternative, indirect methods have been investigated. In the indirect approach, the oleogelation process involves two separate stages and includes techniques such as the emulsion template, foam template, and solvent exchange method [19]. The emulsion template technique involves, first, the formation of an oil-in-water emulsion, after which the emulsion undergoes a drying process to remove the water phase and prepare the oleogel network [20]. Similarly, in the foam template technique, the removal of the aqueous phase leads to a porous polymer structure that entraps the liquid oil and forms an oleogel [21]. On the other hand, in the solvent exchange method, a secondary solvent is used to introduce gelators. Once the gelators are adequately dispersed, the solvent is removed by evaporation, resulting in the stabilization of the oil phase [22].

#### 2.1.1. Oleogelators

An ideal oleogelator must be edible, economical, adaptable, effective (able to structure lipids at relatively low concentrations), and capable of forming structures with comparable physical properties to the lipid material it is intended to replace. Such properties may include hardness/plasticity at a specific temperature, spreadability, a specific melting point, and a specific melting profile [23].

Various oleogelators have been used to structure different oils through the direct approach, such as monoglycerides (MGs), diglycerides (DGs), stearic acid (SAC), and different waxes. For example, MGs have been used in various concentrations to structure canola oil [6,24,25,26], corn oil [27,28,29,30], soybean oil [31], sunflower oil [13,32,33], and olive oil [34,35,36]. Binary gelator systems have also been investigated, such as combinations of glycerol monostearate (GMS) with rice bran wax (RBW) in corn oil [37] and soybean oil [38,39] or GMS with beeswax (BW) in soybean oil [11,40]. Also, a mixture of MGs and DGs, along with RBW, has been used for the preparation of the oleogel phase in gelatin (GEL) bigels [41]. Moreover, SAC has been used as an oleogelator in soybean oil in different bigel systems with [12,42,43] or without the addition of soy lecithin (SL) [44,45]. Different waxes have been selected as oleogelators of the oleogel phase of bigels, such as candelilla wax (CW) [46,47] and BW in canola oil [48], corn oil [29,49], medium-chain triglycerides (MCT) oil [50], sunflower oil [51,52], sesame oil [53], and grape seed oil [54]. Moreover, RBW was used to structure soybean oil [41,55] and high oleic soybean oil oleogels [56], while carnauba wax (CBW) has been used to form sunflower oil and olive pomace oil oleogels [57,58]..

The use of polymeric oleogelators for the formation of the oleogel phase in bigels has also been evaluated. For example, ethylcellulose (EC) [59] and whey protein isolate (WPI) [60] oleogels were produced for the preparation of food-grade bigels.

#### 2.1.2. Oils

The type of oil (acting as the solvent in the oleogel system) also significantly affects the thermal, rheological, and mechanical properties, as well as the nutritional characteristics of the produced oleogels and, in turn, the final properties of the bigels. The chemical characteristics of the oil phase (such as the dielectric constant, polarity, and viscosity of the oil) are mainly determined by the saturated/unsaturated fatty acids (SFA/UFA) ratio, the length of the fatty acid chains, the conformation of the triglycerides (TGs) chains, and the presence of polar/non-polar functional groups [61]. Martins et al. [62] reported that the composition of TGs in the used oils is an important parameter for tailoring the properties of oleogels. A variety of vegetable oils has been used in the oleogel phase of bigels, including canola oil [6,24,25,46,47], corn oil [27,28,29,30,37], soybean oil [31,42,63,64], sunflower oil [32,51,59,65], sesame oil [53] and olive oil [34,35,36]. Also, oils, such as coconut oil, avocado oil, and pomegranate oil, that contain bioactive compounds, have been evaluated as the lipid phase of bigels [66,67]..

### 2.2. Hydrogels Used for Bigel Preparation

Polymeric hydrogels are aqueous networks of polymer chains cross-linked via chemical or physical bonds. Hydrogels are formed using macromolecules with hydrophilic groups like -OH, -COOH, -SO_3_H, -CONH-, and -CONH_2_- that either grafted to or embedded in their polymeric backbones [68]. Generally, hydrogels are described as intermediate entities which exhibit both elastic (solid-like) and viscous (liquid-like) features. Due to the presence of hydrophilic regions and groups, hydrogels have the ability to absorb and retain amounts of water that are several hundred times their dry weight within the 3D network they form [69]. Hydrogels play significant roles in food formulations, primarily as thickeners and stabilizers [70]. Also, hydrogels are used in the development of edible food packaging materials, delivering nutraceuticals while ensuring their bioavailability, regulating the intake of calories from foods, and structuring foods to attain desired sensory textures. Parameters that affect the hydrogelation of formulations include the temperature, pH, ionic strength, and gelling agent concentration [71].

#### Hydrogelators

For the preparation of food-grade bigels, hydrogels have been formulated from natural polymers, such as polysaccharides and proteins. Vershkov and Davidovich-Pinchas [50] and Zheng et al. [72] prepared xanthan gum (XG) hydrogels, while other researchers [11,13,40,73] have utilized gellan gum (GG) hydrogels as a part of bigels. Additionally, bigels with guar gum (GGM) [26], wheat starch (WS) [59], sweet potato (SPS) and chayote tuber starch (CTS) [31,48], sodium alginate (SA) [50], chitosan [74], and tapioca (TAP) [52] have also been used. Composite hydrogels with κ-carrageenan (KC) and locust bean gum (LBG) [33], KC and GEL [35], KC and XG [49], KC and SA [38], or konjac glucomannan (KG) and GEL [45] have been used for bigel production. Moreover, polymers such as carboxymethyl cellulose (CMC) [66,67] and hydroxypropyl methylcellulose (HPMC) [63] have also been utilized in bigel preparation. WPI [12,24,42,43,53,58], whey protein concentrate (WPC) [24], collagen (COL) [57,58], and GEL [6,27,32,75,76,77] have also been exploited to structure the hydrogel phase of edible bigels.

### 2.3. Bigel Preparation Techniques

A deep understanding of bigel processing parameters allows for the customization of bigel properties to provide desirable characteristics for use as solid fat replacers or as carriers of bioactive compounds. First, as discussed above, the oleogels and hydrogels are prepared separately using various oleogelators and hydrogelators, respectively. Several current studies have focused on the conditions of the mixing of the oleogel with the hydrogel phase to form a bigel under mechanical stirring at a pre-determined mixing rate (Table 1). Recent work done by Vershkov and Davidovich-Pinhas [47] focused on the effect of the homogenization temperature on bigels’ properties and characteristics.

#### 2.3.1. Cold Homogenization

The process of cold homogenization necessitates an extended period of mixing or a high mixing rate to attain a homogeneous system [13]. The mixing of cold, pre-set GMS oleogels and SPS hydrogels at 25 °C at 10,000 rpm for 3 min has been suggested by Barroso and coworkers [31]. The rapid homogenization process resulted in the efficient dispersion of the oleogel phase in the hydrogel phase, forming the bigel structure. In contrast, lower mixing rates require longer processing times to ensure the homogeneity of the system. For example, BW oleogels were mixed with SA hydrogels at 600 rpm at 22 °C for 45 min [50]. By adjusting the mixing speed and time of the two phases, the final texture and stability of the bigel can be significantly affected. 

#### 2.3.2. Hot Homogenization

Bigels can also be formed by applying hot emulsification to blend the oleogel phase with the hydrogel phase [35,38,39,45,52,64]. This methodology typically requires a shorter mixing time but a longer setting period for the bigels to solidify, in contrast to cold homogenization. For instance, Samui et al. [6] used the hot emulsification method to prepare bigels based on a GEL hydrogel and a GMS oleogel, with the incorporation of SL as a surfactant and glycerol as a co-surfactant. Also, in the study by Cho et al. [63], a hot RBW oleogel was homogenized with a hot GEL hydrogel at 85 °C either for 20 s at 24,000 rpm or 30 s at 13,000 rpm. This approach facilitates the dispersion and integration of the oleogel and hydrogel phases, leading to the formation of stable bigel structures which can be applied in food products.

## 3. Bigel Characterization

**Figure 1:**
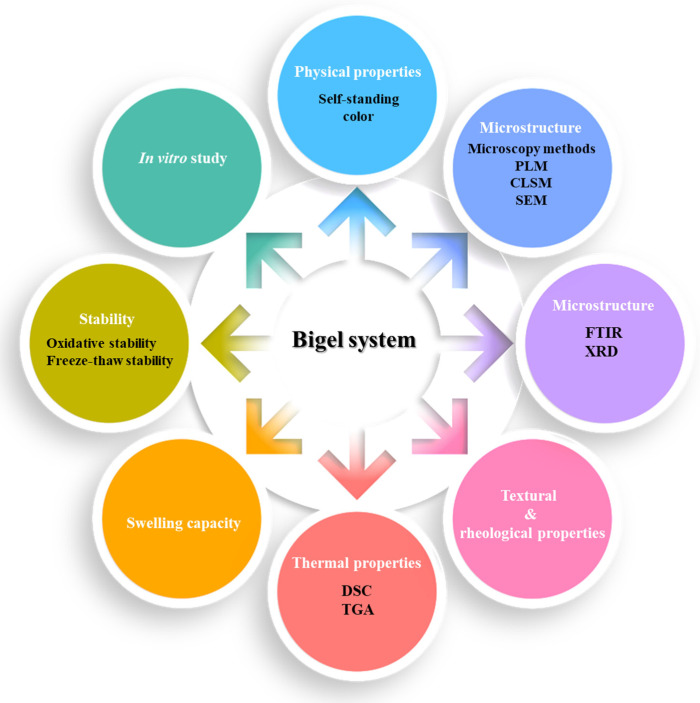
The most used characterization techniques for bigel systems.

Figure 1 presents the most commonly used techniques for the characterization of a bigel system, including the evaluation of its physical properties, examination of the microstructure to determine its morphology, structural organization, and chemical composition, textural and rheological analysis to assess its mechanical and viscoelastic properties, differential scanning calorimetry (DSC) to assess its thermal behavior, etc. Each technique, along with findings from studies on food-grade bigel systems, is discussed in the sections below.

### 3.1. Physical Properties

Biphasic formulations that exhibit structural matrix continuity and a self-standing ability are considered successfully formed bigels. The tube inversion method has been mainly used to evaluate the self-standing ability of bigels [31,33,35,72]. The formation, appearance, and color of a bigel system are related to the composition and mixing ratio of both phases. The color of the bigel is a crucial factor for various food applications, as it may significantly impact the appearance of the final product, affecting the consumer perception and acceptance. In the study conducted by Bollom et al. [42], it was observed that the studied bigels displayed an opaque and off-white visual appearance, irrespective of the composition of their protein or water content. An increase of the oleogel proportion resulted in bigels with a more yellowish hue and that were greasier to the touch, which can be attributed to the presence of olive oil [35,78]. On the contrary, higher hydrogel ratios resulted in bigels with a whiter color [26]. According to data obtained from the evaluation of color parameters using a colorimeter, the lightness (L* value) and redness (a* value) decreased, while the yellowness (b* value) increased, for bigels structured with higher CBW oleogel fractions [58]. The incorporation of KC or WPC80 hydrogels in MGs oleogel resulted in a light color and smooth texture [24]. Also, Samui et al. [6] reported that, depending on the selected composition, bigels may exhibit a white opaque appearance and a variety of textures, from soft “mayonnaise” to hard “butter”. According to Saffold and Acevedo [64], the addition of MGs and DGs to RBW/GEL bigels contributed to softer and more malleable bigels without visual differences compared to the control. Bigels that were produced with GG as the hydrogelator and GMS/BW as the oleogelators remained stable without alterations in color and appearance for 3 months [11], while those produced with EC with XG and GGM were visually stable for 2 months [78].

### 3.2. Microstructure

#### 3.2.1. Microscopy Methods

**Figure 2:**
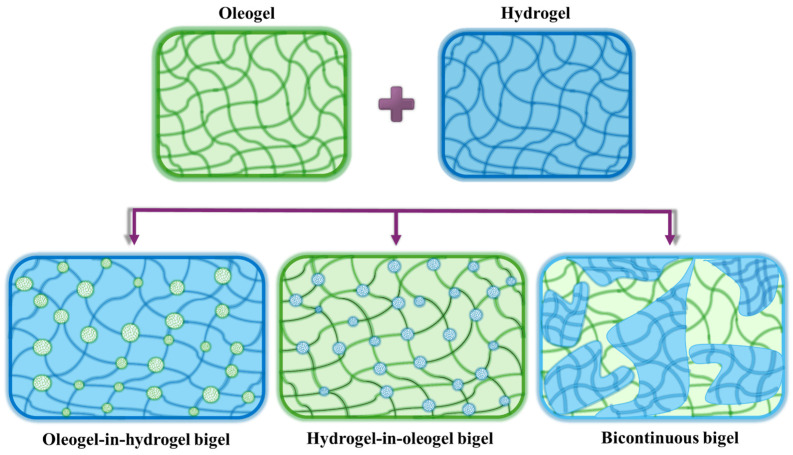
Schematic illustration of the three types of bigels that were formed by mixing an oleogel and a hydrogel phase in different ratios.

The characteristics of a bigel system are contingent upon the structural distribution of the oleogel and hydrogel phases within the bigel matrix, as well as the average size of the dispersed phase droplets. The types of formed bigels have been investigated in many studies by examining their microstructures with techniques such as optical and polarized light microscopy (PLM) [36,77], confocal laser scanning microscopy (CLSM) [37,39], and scanning electron microscopy (SEM) [28,49]. In general, bigels can be categorized as oleogel-in-hydrogel, hydrogel-in-oleogel, or bicontinuous systems [79] (Figure 2).

Oleogel-in-hydrogel systems, where the hydrogel is the continuous phase and the oleogel is the dispersed phase, are possibly the most extensively researched bigel systems in the literature. For example, the formation of an oleogel-in-hydrogel RBW/GEL bigel for all the studied oleogel-to-hydrogel ratios was documented through CLSM imaging [41]. The second type of bigel, known as hydrogel-in-oleogel, comprises a system in which the hydrogel phase is dispersed throughout the continuous oleogel matrix. Microscopy studies confirmed that, as the oleogel ratio increased over 50% in EC/XG/GGM bigels, an inversion in the continuous phase was detected and the type of bigel changed from oleogel-in-hydrogel to hydrogel-in-oleogel [77]. Additionally, other authors have determined the formation of hydrogel-in-oleogel-type bigels using GMS in high oleic sunflower oil with GG, MGs in canola oil with KC and WPC80, and MGs with RBW in corn oil and SA [13,24,37]. The bicontinuous-type bigels are considered as more complex metastable structures, where there is no discrete continuous or dispersed phase due to the co-existence of the two structured phases in the bigel matrix [6,77]. In a study by Zheng et al. [72], very few oil droplets were visible in KC/MGs bigels with a 60% oleogel fraction, with the micrographs showing that the oleogel phase had a dispersal that could be considered as continuous. In addition, a significant percentage of the hydrogel phase was also combined with the oleogel phase and did not form discrete droplets. Thus, such a bigel was considered to form a bicontinuous structure [30,77]. Variation in the oleogel and hydrogel ratios, specifically in the proportions of 30:70, 50:50, and 70:30, in the presence of various emulsifiers, such as MGs and SL, resulted in a significant modification of the structural compositions of the bigels [75]. This transformation involved a shift from the conventional oleogel-in-hydrogel configuration to either a hydrogel-in-oleogel arrangement or a bicontinuous state. Guo et al. [40] reported that, as the oleogel ratio increased from 62% to 70%, the oleogel oil droplets started to aggregate and form a continuous oil phase with a small number of oleogel droplets. Simultaneously, the hydrogel phase underwent a transition from a continuous state to exhibiting block-like regions, and some spherical droplets of hydrogel were also formed, confirming that it was a bicontinuous-type of bigel. When the hydrogel fraction decreased below 30%, a hydrogel-in-oleogel structure prevailed [40]. Several researchers have studied the impact of the oleogel and hydrogel ratio on the microstructures of bigels. Micrographs of bigels revealed that the oil droplets became greater in size, more asymmetrical in form, and more tightly packed inside the hydrogel matrix as the oleogel percentage increased [35,41,77]. Similarly, smaller-sized GMS oleogel particles were obtained by increasing the KC/LBG hydrogel content in bigels. Likewise, an increase in polymer concentration led to the formation of smaller, more uniformly distributed oleogel oil droplets, thereby enhancing the structural stability of bigels [33]. A comparable observation has been reported for MGs/BW and high-acyl gellan gum (HAGG) bigels with up to a 60% oleogel ratio and using CLSM. Zhu et al. [11] attributed the differences observed in the micrographs of their bigel to the enhanced viscoelastic properties of the system due to the increased quantity of oleogel, which hindered homogenization and resulted in the formation of larger droplets. For hydrogel-in-oleogel bigels (oleogel:hydrogel ratios of 80:20 and 60:40), Chen et al. [63] reported that BW soybean oil oleogel acted as the continuous phase in HPMC bigels, allowing sufficient BW crystal growth to form thin needle-like crystals, similar to those in plain BW–soybean oil oleogels. On the other hand, in bigels with oleogel:hydrogel ratios of 50:50, 40:60, and 20:80, the oleogel phase was identified as the dispersed phase, where the limited size of the oil droplets led to smaller crystalline structures organized in clusters. On the other hand, in the study by de Los Santos-Trinidad et al. [48], a higher oleogel ratio in SPS/CTS and BW bigels resulted in a greater oleogel distribution within the continuous hydrogel phase, resulting in the formation of smaller and more spherical droplets. Also, Habibi et al. [24] documented that the properties of hydrogelators (e.g., KC and WPC80) affect the crystallization of MGs in hydrogel-in-oleogel bigels, given that the same oleogel-to-hydrogel ratio and homogenization settings were used for the bigel preparation.

SEM has been used mostly to examine the surface morphology of freeze-dried bigels and bigel beads [28,51]. The addition of the oleogel phase in hydrogel resulted in the formation of continuous networks characterized by dispersed particles [51]. The bigel beads containing 10% BW oleogel exhibited a rough surface and an irregular form according to SEM images. Also, the degree of protuberance decreased, and the surface became smoother as the oleogel concentration increased [28].

#### 3.2.2. Fourier Transform Infrared Spectroscopy

Fourier transform infrared spectroscopy (FTIR) has emerged as a powerful technique, capable of furnishing valuable insights regarding the functional groups, molecular bonds, and general chemical characteristics of bigels. Through the analysis of their FTIR spectra (typically in the range of 4000–400 cm^−1^), researchers can gain insights into the molecular interactions and structural properties of bigel systems. These observations hold great importance, as they facilitate comprehension of the properties and, thus, the potential applications of the formed bigels. The FTIR spectrum of a bigel system is sensitive to changes in bigel composition, mixing speed, preparation methods, and the concentration of the components. Specifically, the preparation technique can affect the molecular interactions, phase distribution, and chemical environment within the bigel matrix. For example, hot emulsification can cause phase transitions or crystallization in certain components, and these can be observed as peak shifts or changes in the FTIR spectrum. In many studies, the impact of the various oleogel-to-hydrogel ratios on the FTIR spectra of bigels was observed through reductions in hydrogen bonding and alterations in the intensities of specific peaks [75,77]. In one study, as the oleogel ratio increased, the intensity of the signals at ~2920, 2850, and 1465 cm^−1^, which represent the stretching vibrations of C-H or C=H from the fatty acyl chains in the vegetable oils phase, also increased [33]. Furthermore, the absorption signals representing C=O stretching vibrations in the oil phase exhibited an increase as the oleogel ratio increased. Zhu et al. [11] reported that some of these signals were entirely lost in bigels due to a low oleogel content (<20%). In contrast, a higher hydrogel ratio in the bigel system was related to an increase in the intensity of the absorption peak in the broad band (3690–3000 cm^−1^), which is associated with O-H stretching vibrations [11,30]. Similar changes were observed by Saffold and Acevedo [41] for RBW/GEL bigels. Chen et al. [63] determined the type of HPMC/BW bigels as oleogel-in-hydrogel, identifying an increase in the absorption intensity of two peaks of HPMC, around 3480 and 1056 cm^−1^, in the bigels’ spectra. Overall, the researchers did not detect major shifts or new peaks resulting from the interaction between the two structured phases in the studied bigels. This confirms the absence of a chemical affinity between the structured phases and underlays the dominance of the physical interactions in the formation of bigels [13,48].

#### 3.2.3. X-Ray Diffraction

X-ray diffraction (XRD) analysis can be conducted on bigel systems to describe the nature of the formed structures by identifying amorphous or crystalline regions. Various studies have documented the crystallinity of different bigel systems using an X-ray diffractometer equipped with a Cu tube. XRD analysis has suggested that bigels exhibit different crystalline structures depending on the type of the oleogelator [29]. Bigels formed with 50% MG oleogels had the highest crystallinity [30]. Ghorghi et al. [54] noted negligible differences in the diffraction patterns among bigels with various BW oleogel-to-SA hydrogel ratios and confirmed that the oleogel phase constituted the crystalline structure of the studied bigel. According to Quilaqueo et al. [46], the formed bigels in their study were semi-crystalline solids, characterized both by crystalline and amorphous components. The incorporation of GG hydrogel in bigels decreased the crystallinity of the system due to its amorphous state [13]. In contrast, XRD analysis in KC with LBG and GMS bigels revealed that they had a mainly amorphous structure, demonstrating a loss of crystallinity compared to the gelator compounds and oleogels used to form the bigels [33]. Similar findings were also reported earlier by Martins et al. [50] for SA with BW bigels, as no polymorphism was observed for all examined oleogel-to-hydrogel ratios.

### 3.3. Textural and Rheological Properties of Bigels

Texture profile analysis (TPA) is conducted to evaluate the textural attributes of bigel systems. The mechanical properties of bigels can be customized and optimized depending on the desired attributes of the final product and the specific processing conditions applied. The mechanical characteristics of bigels, such as hardness, spreadability, and adhesiveness, were found to depend on the bigel composition [6,13], as well as the processing parameters [26]. Additionally, rheology has been used to provide information about the structure and viscoelasticity of bigels and their resulting suitability for applications in the food industry.

#### 3.3.1. Oleogel-to-Hydrogel Ratio

The majority of research on biphasic systems has identified a clear correlation between the mechanical characteristics of bigels and the oleogel-to-hydrogel ratio used. For this reason, a more extensive discussion regarding the impact of modulating the ratio of the two phases is provided in this section. Typically, mechanical properties such as the storage modulus, stiffness, and fracture stress of the bigels have been improved with higher contents of oleogel. Also, better mechanical characteristics were reported when the type of bigel was modified from oleogel-in-hydrogel to bi-continuous or hydrogel-in-oleogel [30]. The effect of the ratio of the two structured phases on the textural properties of GEL and GMS bigels was investigated by Samui et al. [6]. The findings of the study demonstrated a noticeable increase in the hardness of the bigel when the oleogel ratio was increased up to 60% [6]. Also, higher firmness values were observed for WPI bigels with 70% oleogels, demonstrating a clear positive correlation between the firmness of the bigels and their oleogel proportion [60]. A significant increase in the stiffness and fracture stress of MGs/KC bigels has been noted as the MGs oleogel percentage increased, while a strong linear correlation between the two examined parameters was also identified [30]. An increase in the EC oleogel concentration led to a subsequent increase in the hardness and gumminess, while it decreased the adhesiveness, springiness, stringiness, and chewiness of the obtained bigels [78]. Similar findings have been reported by Zheng et al. [30] and Zhu et al. [11]. Habibi et al. [24] noted that the addition of 20% KC hydrogel in bigel weakened the structural integrity of the MGs crystalline network in the oleogel, thus reducing the final hardness of the bigel. According to Han et al. [37], increased GMS/RBW oleogel fractions in bigels led to an increasing trend in their cohesiveness, consistency, and viscosity index. These findings were consistent with previous research on MGs/BW with HAGG bigels [11]. Also, in the study conducted by Mata-Mota et al. [26], it was observed that the firmness of the obtained bigels significantly decreased when the GGM hydrogel proportion was increased from 63% to 87%. The results reported by other researchers further support the impact of the oleogel-to-hydrogel ratio on the textural profile attributes of bigels [33]. However, no discernible changes were detected in the springiness parameter of the bigels, indicating a high recovery capability of the system [33]. On the other hand, Martins et al. [50] found an opposite trend for SA/BW bigels with oleogel-to-hydrogel ratios varying from 50:50 to 99:1, as a reduction in the hardness and spreadability values was observed when increasing the oleogel proportion. Also, bigels with a 65:35 CW oleogel-to-XG hydrogel ratio were, on average, more cohesive compared to the other examined ratios. Interestingly, the findings of Vershkov and Davidovich-Pinchas [47] pointed out a weak correlation between bigel mechanical properties and the oleogel-to-hydrogel ratio, in contrast with the aforementioned studies.

Similarly, the most important variable influencing the rheological characteristics of bigel systems is the oleogel-to-hydrogel ratio. In one study, bigel formulations exhibited a non-Newtonian shear behavior with pseudoplastic characteristics, as demonstrated by a reduction in viscosity (η) and an increase in shear stress (τ) when the shear rate (γ) was increased [46]. Several studies have documented that, in bigel systems, a larger oleogel fraction leads to a higher η [49,51,63]. Additionally, rheological analysis has indicated a solid-like rather than a viscous behavior for bigels, with the storage modulus (G′) having higher values than the loss modulus (G″) in the linear viscoelastic region (LVR), without a cross-over point between G′ and G″. These results were confirmed for MG-BW and HAGG bigels [11], EC and XG/GGM bigels [77], KC and MG bigels [30], BW with agar/XG or GEL/XG bigels [51], and RBW and SA/KC bigels [34], identifying the synergistic effect of the two structured phases on the viscoelastic properties of final bigels and the formation of a network structure with a higher density. At 20 °C, bigel formulations with RBW and GEL showed frequency independence and a more solid than liquid (G′ > G″) character, regardless of the oleogel-to-hydrogel ratio. Additionally, a crossover point between G′ and G″ was not found in the angular frequency range of 0–100 rad/s, suggesting that all bigel formulations may be categorized as gels in the examined frequency range, and that they have enhanced mechanical properties compared to their individual oleogel and hydrogel phases [41]. Moreover, the G′ values of bigels was shown to increase with increasing ratios of GMS/BW-based oleogels; this may be due to the rigid structures of the studied oleogels. Fasolin et al. [13] used GG and high-oleic sunflower oil with GMS to form bigel. They concluded that the obtained bigels exhibited superior strength compared to the original oleogels and hydrogels, displaying higher values of complex moduli (G*) and lower phase angle δ values (defined by tanδ = G″/G′), particularly when the oleogel content was increased, indicating a synergistic effect between the two phases. Also, the results of the mechanical parameters were in agreement with rheological results, as the bigels exhibited high levels of hardness, shear work, and adhesion work [13]. A reduction in the LVR of bigels was observed with an increase in the oleogel content corresponding to the transition of the type of bigel from oleogel-in-hydrogel to hydrogel-in-oleogel [78]. Similar results have been reported in another study where the linear viscoelastic behavior of the bigels was mainly affected by the continuous oleogel phase in hydrogel-in-oleogel bigel [24]. Bigels containing a greater proportion of hydrogel phase exhibited enhanced rheological characteristics due to having a higher critical strain. Such gels have the ability to withstand greater shear forces, providing a more desired semi-solid behavior compared to plain oleogels [42]. Additionally, Zheng et al. [25] reported that the G′ slightly increased as the frequency increased, suggesting that bigels with greater hydrogel proportions had higher η values and less elastic characteristics than bigels with higher oleogel content.

#### 3.3.2. Type and Concentration of the Gelators

Several authors have reported the effects of different concentrations and types of structurants on the rheological and mechanical properties of bigels. Overall, increasing the gelator concentration results in enhanced mechanical properties of the oleogel or hydrogel, and, consequently, of the bigel. For example, the gel strength of the BW- and GMS-based bigels increased progressively with higher oleogelator levels. The increased hardness of the bigels was attributed to the interaction forces between oleogelator molecules, causing the formation of oleogel networks [29]. Bollom et al. [42] alsο documented that a higher concentration of SL/SAC in the oleogel phase contributed to higher G′ values of the obtained bigels. This observation agreed with the results of Cho et al. [55] and Nutter et al. [38]. Additionally, Lu et al. [27] confirmed that the concentration of the oleogelator affects the hardness and the η of bigel systems. The higher content of GMS in the oleogel phase formed a stronger oleogel and, in turn, resulted in greater mechanical properties of the resultant bigels. Also, in bigels fabricated using oleogels structured with WPI, the firmness increased with increasing WPI levels. The firmness value significantly increased from 40.80 to 110.90 as the WPI level increased from 6% to 8%. This finding suggested that an increase in the WPI level reinforces the gel network of the continuous matrix in oleogel-in-hydrogel bigels, enhancing their mechanical strength [60]. Golodnizky and Davidovich-Pinhas [25] documented that the hydrophilic–lipophilic balance (HLB) value of sucrose esters (SEs) had a notable influence on the rheological properties of bigels. For example, bigels fabricated using SEs with lower HLB values showed higher elasticity and exhibited solid-like behavior in comparison with bigels prepared with SEs that had higher HLB values. This difference in rheological behavior was attributed to the low average droplet size and to the narrow particle size distribution of the bigels, as documented by CLSM [25]. The mechanical properties, such as adhesiveness, spreadability, and hardness, and rheological characteristics of bigels are influenced by the type of hydrogel used [13,51]. Martins et al. [33] documented a significant impact of the polymeric structurants content (KC/LBG) of the hydrogel on the consistency of bigels. Similarly, another study emphasized the significance of the composition of the hydrogel phase on the cohesiveness and hardness of bigels. When KC was incorporated into the GEL hydrogel phase of a bigel system, it resulted in increased hardness and reduced cohesiveness due to the cross-linking of the structurants [35]. Regarding rheological parameters, bigels with agar and XG showed higher G′ and G″ moduli than bigels with GEL and XG. This difference was attributed to the distinct chemical-physical architectures of the used polymers or the more substantial intermolecular interactions between the elements of the bigel matrix [51].

#### 3.3.3. Process Parameters and Storage

The mixing rate and mixing time of the oleogel with the hydrogel also affect the textural characteristics of the resulting bigel. For instance, bigels with MGs and GGM exhibited improved mechanical properties when prepared with a lower mixing time and higher mixing rate [26]. According to Fasolin et al. [13], the mechanical properties of guar gum/GMS bigels were significantly affected by increasing the mixing speed from 500 to 1500 min^−1^ for 10 min. Moreover, the mechanical properties of bigels are also dependent on the storage temperature. Bigels with CW and XG stored at 4 °C showed higher firmness, cohesiveness, and plasticity compared to bigels stored at 25 °C, due to the more organized crystal network of the oleogel phase at this temperature [47]. Also, GEL/GMS bigels that were set at a temperature of 4 °C exhibited greater hardness values compared to bigels kept at 25 °C [6]. Zampouni et al. [36] documented that KC/GEL/MGs bigels exhibited textural stability for up to 14 days at 4 °C. Similarly, other studies reported that storage for up to 90 days did not significantly affect the hardness of GEL/MGs bigels [6].

### 3.4. Thermal Properties of Bigels

Thermal characterization aims to interpret the physical characteristics of bigels as a function of temperature and time under certain conditions through the use of appropriate experimental techniques. An important property of polymeric gelled systems is the temperature-dependent gel–sol (solid-like to liquid-like) transition, which is crucial for food applications. Several studies have examined this transformation using various techniques, such as differential scanning calorimetry (DSC) and thermogravimetric analysis (TGA), to evaluate the thermal transition that occurs during heating and the contribution of bound and unbounded water to this process, respectively.

DSC was used for the thermal analysis of bigels with RBW and GEL in the study by Cho et al. [55]. The thermographs revealed two characteristic endothermic peaks during the heating cycle; one corresponded to the melting of GEL around 35 °C, and the other, a sharp endothermic peak at 66 °C, was attributed to the melting of RBW of the oleogel phase. In RBW/SA/KC bigels, the heating and cooling thermograms, regardless of the oleogel-to-hydrogel ratio, revealed prominent peaks at around 70.0 °C and ~65.6 °C, confirming rice bran wax phase transitions. A similar melting profile has been reported for bigels prepared by combining an SA/KC hydrogel with a RBW oleogel [38]. However, other researchers have suggested that the thermodynamic stability of bigels was enhanced by the presence of HAGG hydrogel, as the studied bigels exhibited higher melting temperatures than plain GMS/BW-based oleogels [11]. On the contrary, Ghorghi et al. [54] documented that the thermal properties of SA/BW bigels were improved by increasing the hydrogel proportions in the biphasic system. The DSC analysis of bigels provided further confirmation regarding the co-existence of two structurally organized phases within the bigel system [35]. In the study by Zheng et al. [72], the melting temperatures of bigels with varying oleogel/hydrogel ratios did not show any significant differences, indicating that the thermal behavior of oleogels with fatty acid mixtures remained stable even after they were mixed with XG hydrogels. This demonstrated that bigels can preserve their oleogel-like thermal properties. Habibi et al. [24] reported that the incorporation of KC/WPC80 hydrogel in MG oleogel in an 80:20 ratio decreased the melting point of the MGs. In contrast, the melting behavior of bigel systems remained unchanged when the size of structured aqueous droplets was reduced by the application of different mixing methods [24]. Additionally, several studies have reported that the enthalpies of the endothermic peaks were higher as the proportion of oleogel in the bigels was increased, due to the extended crystallization [35,72].

### 3.5. Swelling Capacity

Assessing the swelling capacity of bigels can provide valuable information on the way that a bigel performs in the digestion process and whether it can subsequently release incorporated bioactive components. The swelling capacity is the measure of the ability of a bigel to absorb water and provides insights into the bigel’s structure, functionality, and potential applications. According to Zheng et al. [30], a 50:50 oleogel-to-hydrogel ratio exhibited a high swelling capacity in bigels with KC and MGs. A higher ratio of oleogel increased the hydrophobic nature of bigels and hindered water absorption in the biphasic system [30,35,78]. Thus, the most important factor that affected the swelling ratio was the bigel type. When the continuous phase was comprised of hydrogel, the absorption of water was promoted, while, when oleogel was the continuous phase, the penetration of the water was hindered [11]. Also, Kaimal and Singhal [78] reported that a higher hydrophilicity of the hydrogelator facilitated the absorption of water and allowed bigel to swell. Interestingly, a low swelling capacity of bigels was observed when KC with GEL was used as a hydrogelator, due to the formation of a stiff network that did not allow the cross-linked polymeric chains to expand and absorb water [35].

### 3.6. Stability

#### 3.6.1. Centrifugation Stability

The assessment of the solvent holding capacity (SHC) of bigels was employed to evaluate the ability of the bigel matrix to retain a significant amount of solvent when subjected to centrifugation forces (e.g., 10× *g* for 15 min or 9200× *g* for 15 min) at room temperature [6,33]. The results indicated that treatments with GEL hydrogel and GMS oleogels mixed in ratios of 60:40 and 50:50 exhibited full stability under high centrifugal forces [6]. According to the results of a similar study, the extent of solvent retention within the bigel structure demonstrated an enhanced degree of stability and the dominance of the polymeric network of locust bean gum with KC hydrogel, even in bigel treatments with low GMS concentrations [33].

#### 3.6.2. Oxidative Stability

The determination of the oxidative state and stability of bigel systems is crucial for their use as fat replacers, because they could affect the flavor, the aroma, and the nutritional value of food products during manufacturing and storage. The development of oxidation is influenced by several factors, such as the composition and origin of the oil used in the oleogel phase, the ratio of the oleogel to hydrogel, the oleogel and bigel preparation temperatures, the type of the formed bigel, the duration and the temperature of storage, and the addition of pro- and anti-oxidant agents. Several methods have been used for the evaluation of the oxidative stability of bigel systems. Quilaqueo et al. [46] measured the peroxide values (PVs) and p-anisidine values (AVs) to examine the extent of primary and secondary oxidation in bigels, respectively. The authors concluded that the use of CMC hydrogel in bigel did not offer protection against the development of primary oxidation products during the bigel preparation process. However, lower PVs were measured in bigel systems compared to plain BW-canola oil oleogels due to the lower lipid content in bigels. Similar results were also reported by Baltuonytė et al. [57], as bigels structured with GEL and agar showed lower oxidation compared to a mixture of sunflower oil and olive pomace oil after 14 days of storage at 37 °C. Also, other studies have obtained lower PVs during 30 days of storage at 4 °C for SA/BW bigels by elevating the hydrogel content in the biphasic system [54]. Cho et al. [41] reported that the PVs of bigels remained low during storage time (180 days) at 22 ± 1 °C, even when 1 mg Cu^2+/^kg oil was added as a pro-oxidant. Also, the primary and secondary oxidation products were determined in GEL/MGs bigels by measuring the PVs and thiobarbituric acid (TBA) values [6]. Regarding PVs, the bicontinuous GEL/MGs bigels showed a behavior that was dependent on time, temperature, and the hydrogel content of the bigels. According to Samui et al. [6], the hydrogel phase enabled oxygen diffusion to the oleogel phase, promoting the oxidation process. Also, it is interesting to note that the TBA values of bigels exhibited a gradual rise during storage at different temperatures. On the contrary, the study of Quilaqueo et al. [46] reported that the AVs of CMC/BW bigels were lower than those of BW oleogels, indicating that the presence of the hydrogel phase at a 50% ratio provided protection against the development of secondary oxidation products.

#### 3.6.3. Freeze–Thaw Stability

The freeze–thaw stability of bigels is crucial for frozen foods and is directly dependent on the degree of the collapse of the hydrogel network and the formation of ice crystals during freezing. The freeze–thaw stability of bigels has been evaluated by measuring the oil holding capacity (OHC) and water holding capacity (WHC) of bigels after freeze–thaw cycles [29,63]. According to Martins et al. [33], the degree of the oil holding capacity was indicative of the bigel stability. Different oleogelators formed distinct crystalline networks, which, in turn, affected the bigel oil binding capacity [45]. Additionally, the WHC was strongly influenced by both the architecture and the stability of the formed gel network [60]. Generally, the OHC and the WHC have been evaluated using centrifugation for a certain time [29,45]. The OHC of bigels was improved by the incorporation of WPC80 hydrogel into the MGs oleogel matrix [24]. Also, an increase in the homogenization time from 1 to 3 and 5 min also increased the OHC in GEL/MG-based bigels [6]. Yang et al. [29] documented that the type of the formed bigels, the strength of the oleogel phase, and the type of oleogelator affected the freeze–thaw stability. In GMS/KC bigels, both the WHC and OHC increased with an increase in GMS content, due to the formation of stronger oleogel networks and more closely packed oil droplets. On the contrary, in BW/KC bigels, only the OHC significantly increased with an increase in the BW content. However, phase separation of the hydrogel and oleogel was observed after freeze–thawing, indicating the disruption of the interfacial layer due to the growth of ice crystals [29].

## 4. Bigel Applications

Table 2 provides an overview of the current food applications of bigels, highlighting their potential in various sectors of the food industry. For instance, according to the literature, bigels can be used as fat substitutes, edible coatings, or effective delivery systems for bioactive compounds. Also, bigels have been investigated as innovative food matrices and edible food packaging materials, using 3D food-printing technology.

### 4.1. Bigels as Delivery Systems for Bioactive Compounds

Several studies have utilized in vitro digestion protocols to investigate the potential of bigel systems to act as vehicles for the efficient delivery of bioactive substances. For example, GMS/BW and HAGG bigels in various oleogel-to-hydrogel ratios were evaluated for the delivery of lycopene [11]. After gastric and intestinal phase digestion, a higher percentage of lycopene was released from bigels containing a lower oleogel ratio, indicating a slower and more controlled release of the compound. Zhu et al. [11] suggested that the oleogel enhanced the bigel structure, resulting in slower penetration of the gastric fluids into the bigel network, and practically inhibiting the release of lycopene during gastric digestion. On the other hand, the collapse of the hydrogel network during intestinal digestion enabled pancreatic enzymes and bile salts to successfully break down the oleogel, causing the controlled release of lycopene. Kaimal and Singhal [78] developed EC and XG/GGM bigels to study the effectiveness of biphasic systems in the delivery and protection of another carotenoid substance, lutein, through a simulated gastrointestinal tract. The results of this study were in agreement with the results of Zhu et al. [11], as the release of lutein from different bigels exhibited statistically significant variations, showing a greater release of lutein as the hydrogel fraction in the bigel increased to 75%. On the contrary, Zheng et al. [30], who studied the utilization of MG/KC bigels as a delivery system of β-carotene, found that both the release rate and the total (%) β-carotene release were increased for bigels with a higher oleogel ratio. Interestingly, the bigel containing 75% oleogel showed the highest release of β-carotene. This was supported by the fact that, in this type of bigel, the continuous phase was the oleogel, which was readily exposed to the enzymes for lipid hydrolysis. Therefore, based on the above, it could be concluded that the types of gelator used for structuring the oleogel and hydrogel phase could modulate the release profile of each substance more effectively than the ratio of the two phases and the formed type of the final bigel. Also, according to Lu et al. [27], the concentration of the oleogel structurant modified the release of the lipophilic curcumin from GMS/GEL bigel, as the increased concentration of GMS resulted in a significantly reduced rate. On the contrary, when epigallocatechin gallate (EGCG) was encapsulated within the hydrogel phase, the impact of this GMS hydrolysis on the release of EGCG was minimal. The release of EGCG occurred rapidly within the gastric juice, as the hydrogel phase was susceptible to pepsin degradation, and subsequently, the release rate remained relatively constant [27]. These findings support the idea that bigel systems have the ability to simultaneously transport bioactive components with varying polarities. For example, Yang et al. [28] suggested that GMS/SA bigel beads with different oleogel-to-hydrogel ratios can succeed in the co-delivery and protection of curcumin and EGCG. An increase in the oleogel content resulted in a gradual decline in the encapsulation efficacy of EGCG, attributed to the relatively diminished water phase content, in contrast with the encapsulation efficacy of curcumin, which showed an opposite trend. Also, a less dense alginate network with a less rigid configuration facilitated the effective diffusion of EGCG through SA gel microstructures and also improved the release of the bioactive substance. After a 40-day storage test, the retention ratio of loaded curcumin was sufficiently high, indicating that the bigel structure prevented the compound from being degraded [28]. Liu et al. [44] investigated the gastrointestinal digestive release of the hydrophobic substance quercetin from KG/GEL bigels. According to the study, an increase in the KG-to-GEL ratio resulted in a decrease in the bioaccessibility of the quercetin due to the formation of a more compact and cohesive bigel. In the same study, the release of volatile compounds (i.e., limonene) in the oral cavity from the bigel structure was evaluated using an electronic nose. The results demonstrated that the modification of the gel strength of the hydrogel phase led to different volatile release behaviors of bigels, with stronger gels preventing the release of the substances [44].

Moreover, MG/CMC bigel systems have been evaluated concerning the ability to offer protection for bioactive fatty acids of vegetable oils (coconut oil, avocado oil, pomegranate oil) during transit through the in vitro gastrointestinal environment. Throughout all stages of digestion, the concentration of the main bioactive fatty acids in each oil remained higher in bigel formulations in comparison to the corresponding oils [67]. Similarly, bigels with avocado oil were incorporated into yogurt to partially modify the profile of saturated fatty acids in milk fat and achieve a more balanced ratio of saturated and unsaturated lipids. Yogurt containing avocado oil bigel exhibited increased stability throughout the simulated gastrointestinal tract, indicating a greater recovery percentage of oleic acid in comparison with yogurt with avocado oil in a free state. The outcomes derived from the in vitro study demonstrated that these functional yogurts possessed the ability to increase the lipolytic rate in adipocytes, as evidenced by an approximate 20% increase in the glycerol release in yogurts with avocado oil bigel [66].

Bollom et al. [38] studied the capability of a bigel system to protect probiotic microorganisms (i.e., *Bifidobacterium lactis* and *Lactobacillus acidophilus*) during in vitro digestion, and also ascertained the impact of phospholipids on the survival of probiotics. The results showed that the bigel structure that results from the addition of the gelators (SL/SAC and WPC80) is crucial for the survival of the probiotics through the gastric phase. In contrast, the same study showed that the addition of phospholipids in the bigel did not provide significant protection to the viability of probiotics during digestion because of their enzymatic breakdown by digestive enzymes. Similarly, other studies have evaluated bigels using the aforementioned gelators for the protection of probiotics in two yogurt products, indicating that the bigel structure enhanced the probiotic viability [12].

### 4.2. Bigels as Fat Substitutes

#### 4.2.1. Meat Products

Bigel systems were incorporated as fat substitutes in coarse-ground fully cooked sausages [56]. The bigels were composed of a RBW oleogel and GEL hydrogel, and were mixed in 70:30 and 60:40 ratios. In this study, the authors reported that the bigel remained stable after all the preparation stages of the sausages and the bigel with a 70:30 oleogel-to-hydrogel ratio exhibited a higher resemblance to the characteristics of pork fat. In an attempt to improve the nutritional properties of semi-dry sausages, Zampouni et al. [36] used two different hydrogel-in-oleogel bigels composed of olive oil oleogels (20%) and KC or KC plus GEL hydrogels (80%) for the partial replacement of pork backfat. The weight loss, moisture content, and water activity were shown to differ significantly between the sausages with fat replacement and the control. These variations were ascribed to the bigels’ composition, the applied dehydration conditions, and the bigels’ greater initial moisture content. Analysis of the textural characteristics of the semi-dry sausages revealed that the fat replacement did not affect the textural attributes of the sausages. Ghiasi and Golmakani [59] reported that EC and starch bigel with a 75% oleogel fraction exhibited excellent performance as a fat substitute in low-fat burgers. According to the results, all the low-fat burgers had superior cooking characteristics compared to the animal fat control burgers, especially when the replacement percentage was higher. The replacement of up to 50% animal fat with bigel resulted to low-fat burgers with acceptable sensory properties, even though the addition of bigel increased lipid oxidation in the burgers. Also, bigels with KC, GEL, and MGs were used to replace animal fat in fermented sausages. The addition of the bigel did not affect the water activity and microbial populations of the produced sausages. Interestingly, the color, texture, juiciness, flavor, taste, and overall acceptability of the sausage treatments did not significantly differ, according to consumer sensory evaluation studies [34].

#### 4.2.2. Bakery Products

Recently, Nutter et al. [38] assessed the complete substitution of solid fats in a high-fat (30% *w*/*w*) short dough product using plant-based bigels. Bigels were formulated using 20% SA/KC hydrogel and 80% RBW soybean oil oleogel, with or without the addition of MGs. The findings of the study revealed that the obtained bigels effectively imitated the functionality of butter and shortenings by providing the desired shortening effect, leading to the formation of inelastic doughs. The desired fat functionality was preserved even after baking, as the bigel cookies exhibited a similar structure, moisture content, and water activity compared to traditional shortbread. Furthermore, the bigel cookies were softer than those made with commercial solid fats such as butter and shortening. Similarly, Quilaqueo et al. [46] evaluated the application of bigel systems comprised of 50% BW oleogel and 50% SA or CMC hydrogel as fat substitutes in cookies. They found that cookies with bigels exhibited similar hardness but significantly higher fracturability compared to cookies made with the original shortening. Regarding changes in the cookie shape after baking, while the width of the bigel cookies remained unchanged, the thickness was significantly increased. In another study, butter was replaced with hydrogel-in-oleogel bigel with RBW, GMS, and SA to enhance the quality characteristics of the bread [37]. The bread produced with bigels exhibited a greater specific volume and softer texture than the bread with butter, regardless of the oleogel ratios in the bigels. Additionally, the researchers concluded that, when the proportion of the oleogel reached 80%, it resulted in bread with an ideal specific volume and the most favorable textural attributes [37]. The above findings indicate the great potential of bigels as a fat replacer in bakery products.

### 4.3. 3D Printing

Additive manufacturing, also known as 3D printing, is an innovative technology that has garnered significant interest across various scientific fields. This manufacturing process involves the layer-by-layer construction of complex solid or semi-solid shapes. The potential of 3D food printing is supported by the ability to create food products that meet consumers’ requirements with both economic and environmental advantages. However, to achieve the formulation of novel printed structures, it is necessary to first understand the rheological properties of the printing materials (food inks) to ensure the feasibility of the printing process [80]. Recently, research has been conducted regarding the development and evaluation of food-grade bigel systems for 3D printing applications. The study by Xie et al. [75] included the fabrication of 3D-printed bigels with a complicated structure which would have the potential to encapsulate catechin and quercetin and enhance the nutritional and functional properties of foods. The main components utilized in the formation of the 3D-printed bigels were GEL hydrogels, CW-based oleogel, and emulsifiers, such as MGs and SL [75]. According to another study by Xie et al. [76], the ratio of oleogel to hydrogel in bigels significantly affected the quality of printing, with better results obtained for higher oleogel ratios. For example, a bigel consisting of MG and SL with an oleogel/hydrogel ratio of 70:30 exhibited a printing performance comparable to that of pure hydrogel, with a smooth surface and good shape retention after the printing process. However, despite its desired shape, there was some oil leakage in the printed object. These 3D printing results indicated that the hydrogel mostly provided the necessary mechanical strength to support the intended shape during the printing process. However, the increased ratio of hydrogel in the bigel could potentially obstruct the printing nozzle, resulting in an unacceptably low printing accuracy of the bigel. Similarly, Chen et al. [63] evaluated the capability of printing by testing the ability of different types of bigels to form structures for replacing conventional solid fats and for the fabrication of food products with specific appearance attributes. Analysis of the printing results revealed that hydrogel-in-oleogels bigels were more suitable for flat stacked models, whereas oleogel-in-hydrogel bigels performed better in models with small-area monolayer structures. Furthermore, TPA tests indicated that the extrusion process had a significant effect on hydrogel-in-oleogel and bicontinuous bigels, but not on oleogel-in-hydrogel bigels.

#### Packaging

The work of Zhai et al. [65] presented an innovative approach to developing colorimetric gas sensors for intelligent food packaging that were based on anthocyanins, using 3D-printable hydrogel-in-oleogel bigel. The bigel used was composed of agar, purple sweet potato anthocyanins, BW, glyceride monooleate, and sunflower oil. Then, the composite film was fabricated by extruding the bigel onto polyvinylidene fluoride (PVDF) film using 3D printing technology. The final PVDF–bigel film was successfully utilized as a volatile amines sensor to monitor meat and fish freshness. While bigel systems offer a lot of advantages such as improved stability and the ability to deliver bioactive compounds, there are several challenges to consider before their use as packaging materials. For example, bigels may exhibit lower mechanical properties than traditional packaging materials or exhibit lower moisture or oxygen barrier properties, which can lead to stability problems and degradation over time.

### 4.4. Other Applications

#### Edible Coating

An earlier study by Kanelaki et al. [26] evaluated the effectiveness of GEL/MGs bigel as an edible coating for sardine fillets. The results suggested that, when bigels loaded with rosemary extract were used as coatings, they delayed lipid oxidation and total volatile basic nitrogen (TVB-N) formation in sardine fillets, but they did not affect microbial growth during storage.

## 5. Conclusions and Future Perspectives

Bigel systems offer numerous benefits over other structured systems, such as oleogels, hydrogels, or even emulsion gels. The characteristics of these biphasic systems are predominantly contingent upon parameters that include the oleogel-to-hydrogel ratio, the type and the concentration of the structural components of each phase, and the methods of fabrication and storage. Therefore, the modification and optimization of the above variables is necessary for the formation of a bigel system that exhibits the necessary and desirable characteristics for use in a food matrix. Nevertheless, it is necessary to determine the resulting microstructure, the mechanical and thermal properties, and the stability of bigels using specific techniques before their utilization for food applications. To date, the versatility of bigel systems has led to the development of applications across a wide range of food products, although still on a lab scale. For instance, bigels have demonstrated potential efficacy as delivery systems of bioactive compounds, fat substitutes in meat, bakery and confectionery products, and edible and biodegradable packaging materials. The encapsulation of various substances (e.g., vitamins, flavorings, antioxidants) in bigels and the assessment of their controlled release and diffusion rate could offer the opportunity for the development of functional and sustainable new food products, meeting current and future consumer demands. Also, the utilization of bigels in 3D printing applications appears to be an innovative approach not only to customize and tailor bigels’ properties but also for the formulation of new products with enhanced functionality. Thus, the future of bigels in the food field is very promising, suggesting potential for a wide range of innovations regarding food formulation, processing, and packaging.

## Figures and Tables

**Table 1:** Examples of edible bigels (composition, oleogel-to-hydrogel ratio, and bigel mixing parameters) that can be used for food applications.

Oleogel (O)		Hydrogel (H)	O:H Ratio	Bigel Mixing Parameters	Reference
Edible Oil	Oleogelator	Hydrogelator			
Canola oil	MGs * (10%)	KC (4%) WPC 80 (15%)	80:20	4000 rpm at 90 °C for 2 min Sonication at 90 °C for 2 min 10,000 rpm for 2 min + Sonication at 90 °C	[24]
	MGs (0–35%) SL (2–20%)	GEL (0.5–3%) Glycerol (5–15%)	20:80, 30:70, 40:60, 50:50, 60:40, 70:30, 80:20	1600 rpm at 70 °C for 3 min	[6]
	MGs (25%) SL (1.6%) SEs (2%)	GEL (1%) Glycerol (5%)	50:50	O:70 °C, H:90 °C 16,000 rpm for 1 min	[25]
	CW (8%) SEs	XG (0.5%)	55:45, 65:35, 75:25, 85:15	20,000 rpm at 30–60 °C for 2.5 min	[47]
	MGs (10%) PGPR (1.5%)	GGM (1%) Tween 60 (1.7%)	67:33, 83:17, 58:15	600/800 rpm for 20 min	[26]
	BW (5%)	SPS (10%) CTS (10%)	30:70, 40:60, 50:50	Sonication at 90 °C for 2 min	[48]
Corn oil	GMS (4–8%) BW (4–8%)	KC (0.75%) Tween 20 (0.5%)	50:50	300 rpm at 45 °C	[29]
MCT oil	BW (3%, 6%)	SA (2%)	50:50, 80:20, 90:10, 95:5, 99:1	600 rpm at 22 °C for 45 min	[50]
Soybean oil	SL, SAC (7:3, 20%)	WPC 80 (15–25%)	30:70, 50:50, 70:30	23,000 rpm for 3 min	[42]
	RBW (10%)	GEL (5, 7, 10%)	20:80, 30:70, 40:60, 50:50	24,000 rpm at 90 °C for 2 min	[41]
	RBW (7.5%)	GEL (7%)	40:60, 50:50, 60:40, 70:30	24,000 rpm at 85 °C for 20 sec 13,000 rpm at 85 °C for 30 sec	[55]
	RBW (10%) MGs/DGs (0.5–3%)	GEL (7%)	60:40, 70:30, 80:20	24,000 rpm for 1 min	[64]
	RBW (9%) MGs (0–2%)	SA (1%) KC (0.5%)	70:30, 80:20	15,000 rpm at 85 °C for 1 min	[38]
	GMS (8%)	WPS (8%)	20:80, 30:70, 40:60, 60:40, 70:30, 80:20	10,000 rpm at 25 °C for 3 min	[31]
	SAC (3%)	KG/GEL (2.25%)	20:80, 30:70 40:60, 50:50	9000 rpm at 75 °C for 3 min	[45]
Sunflower oil	GMS (5–15%)	GG (1–1.5%)	20:80, 35:65, 50:50, 65:35, 80:20	500–1500 rpm at 25 °C for 10 min	[13]
	GMS (5–20%)	KC/LBG (1:1, 0.5–2.5%)	10:90, 20:80, 30:70, 40:60, 50:50	2000 rpm for 10 min	[33]
	Fatty acids mixture (10%)	XG (1%)	40:60, 50:50, 60:40, 70:30, 80:20	14,000 rpm at 85 °C for 3 min	[72]
	BW (6%)	TAP (5–10%)	25:75, 40:60, 50:50, 60:40, 75:25	14,000 rpm at 85 °C for 1 min	[52]
	WPI (40 mg/mL)	WPI (60, 80 mg/mL)	30:70, 50:50, 70:30	800 rpm at 24 ± 2 °C for 10 min	[60]
Sunflower oil Olive pomace oil	CBW (8%) SL (0.5%)	COL (40%, 60%)	40:60, 50:50, 60:40	11,000 rpm at 85 °C for 3 min	[58]
Olive oil	MGs (15%)	GEL (8–12%) KC (1%)	20:80, 40:60	300 rpm at 50 °C or 80 °C for 15 min	[35]
Olive oil Coconut oil	MGs Ps	GEL	40:60, 60:40	600 rpm at 50 °C for 15 min	[77]

* BW: beeswax, CBW: carnauba wax, COL: collagen, CTS: chayote tuber starch, CW: candelilla wax, DGs: diglycerides, GEL: gelatin, GG: gellan gum, GGM: guar gum, GMS: glycerol monostearate, KC: κ-carrageenan, MCT: medium-chain triglycerides, MGs: monoglycerides, PGPR: polyglycerol ricin alkyd ester, Ps: phytosterols, RBW: rice bran wax, SA: sodium alginate, SAC: stearic acid, SEs: sucrose esters, SL: soy lecithin, SPS: sweet potato starch, TAP: tapioca starch, XG: xanthan gum, WPC: whey protein concentrate, WPI: whey protein isolate.

**Table 2:** Bigel systems (composition, oleogel-to-hydrogel ratio, and bigel mixing parameters) and their current application in food products.

Application		Oleogel (O)		Hydrogel (H)	O:H ratio	Bigel Mixing Parameters	Reference
		Edible Oil	Oleogelator	Hydrogelator			
Delivery system	Bioactive fatty acids	Coconut oil Avocado oil Pomegranate oil	MGs * Tween 80	CMC (2%)	Non reported	18.000 rpm for 1 min + sonication for 1 min	[67]
	Bioactive fatty acids in yogurt	Avocado oil	MGs+DGs Tween 80	CMC (2%)	Non reported	18.000 rpm for 1 min + sonication for 1 min	[66]
	β-Carotene	Corn oil	MGs (20%)	KC (1.5%)	25:75, 40:60, 50:50, 60:40, 75:25	300 rpm at 80 °C for 20 min + 500 rpm at 80 °C for 2 min	[31]
	Curcumin EGCG	Corn oil	GMS (4–8%)	GEL (2.5%) Tween 20 (0.5%)	50:50	300 rpm at 60 °C for 20 min	[27]
	Curcumin EGCG in bigel beads	Corn oil	GMS (10%)	SA (1%) Tween 20 (0.5% in bigel)	10:90, 15:85, 20:80, 25:75, 30:70	500 rpm	[28]
	Lutein	Sunflower oil	EC (15%)	XG GGM (1:1, 1.5%)	25:75, 50:50, 75:25	10,000 rpm at 75 °C for 5 min	[78]
	Lycopene	Soybean oil	GMS (1%) BW (1%)	GG (0.3%)	10:90, 20:80, 30:70, 40:60, 50:50, 60:40	10,000 for 1 min	[11]
	Probiotics	Soybean oil	SL, SAC (1:1, 20%)	WPC 80 (20%)	80:20	13,500 rpm at 85 °C for 2 min	[26]
	Probiotics in yogurt	Soybean oil	SL, SAC (1:1, 16%)	WPC 80 (20%)	75:25	13,500 rpm at 85 °C for 2 min	[12]
	Quercetin	Soybean oil	SAC (2.5%)	KG, GEL (2.25%)	Non reported	9000 rpm at 75 °C for 3 min	[44]
Fat substitution	Beef burger	Sunflower oil	EC (10%)	WS (10%)	25:75, 50:50, 75:25	2000 rpm for 2 min + 2500 rpm for 2 min	[59]
	Coarse-ground sausages	High oleic soybean oil	RBW (7.5%)	GEL (7- 8%)	70:30, 80:20	24,000 rpm for 1.5 min	[56]
	Fermented sausages	Olive oil	MGs (15%)	GEL (10%) KC (1%)	20:80, 40:60	600 rpm at 80 °C for 15 min	[34]
	Semi-dry sausages	Olive oil	MGs (15%)	GEL (4%) KC (2%)	20:80	300 rpm at 80 °C for 15 min 12,000 rpm for at 80 °C 15 min	[36]
	Bread	Corn oil	GMS (1%) RBW (2%)	SA (2%)	20:80, 30:70, 40:60, 50:50, 60:40	10,000 rpm at 85 °C for 3 min	[37]
	Cookies	Canola oil	BW (10%)	CMC SA (3%)	50:50	2500 rpm at 30 °C	[46]
	Shortbread	Soybean oil	RBW (9%) MGs (0–2%)	KC (0.5%) SA (1%)	80:20	15,000 rpm for 1 min at 85 °C	[38]
	Butter spread	Sesame oil	BW (10%)	SA (3%) WPC 80 (25%)	Non reported	900 rpm for 2 min	[53]
	Chocolate	Grape seed oil	BW (20%)	SA (2%)	99:1, 95:5, 90:10	600 rpm for 45 min	[54]
3D printing		Corn oil	BW (15%)	KC XG (1:1, 1.5%)	20:80, 30:70, 50:50, 70:30, 80:20	3000 rpm for 2 min + 6000 rpm for 3 min	[49]
		Soybean oil	BW (10%) PGPR (1%)	HPMC (3%)	20:80, 40:60, 50:50, 60:40, 80:20	8000 rpm at 85 °C	[63]
		Soybean oil	BW (10%) GMS (2%)	GG (3%)	30:70, 40:60, 50:50, 60:40, 65:35, 70:30, 80:20	8000 rpm at 80 °C for 3 min + 100 rpm for 10 min	[40]
		High oleic acid sunflower seed oil	CW (3%), MGs (1%), PGPR (1%), SL (1%)	Fish GEL (5%)	30:70, 50:50, 70:30	10,000 rpm for 1 min	[76]
		Sunflower oil	BW (5%)	GEL (10%) XG (1%) Agar (15%)	5:95, 10:90, 20:80	2000 rpm at 50 °C for 10 min	[51]
3D printing	Delivery system of quercetin	High oleic acid sunflower seed oil	CW (3%) MGs (1%) SL (1%)	Fish GEL	30:70, 50:50, 70:30	10,000 rpm for 1 min	[75]
	Food packaging	Sunflower oil	BW (6%) GMS (2%)	Agar (0.5–2%)	90:10, 80:20, 70:30, 60:40	12,000 rpm for 1 min	[65]
Other applications	Edible coating with rosemary extract	Sunflower oil	MGs (15%)	GEL (10%)	20:80	300 rpm min at 70 °C for 15	[32]
	Spread with lingonberry pomace	Sunflower oil Olive pomace oil	CBW (10%) SL (0.5–1.5%)	COL (15.6%) GEL (5%) Agar (2.5%)	60:40	15,000 rpm for 2 min at 85 °C	[57]
	Cream analogue	MCT oil	GMS Cinnamaldelhyde Span 80 (0–0.9%)	Chitosan	20:80, 33:67, 40:60	200 rpm for 20 min	[74]
	Foam-based bigels	Soybean oil	BW (10%) GMS (2%)	GG (1.2%)	20:80, 30:70, 40:60, 50:50, 60:40, 70:30, 80:10, 90:10	8000 rpm for 2 min	[73]

* BW: beeswax, CBW: carnauba wax, CMC: carboxymethyl cellulose, COL: collagen, CW: candelilla wax, DGs: diglycerides, EC: ethylcellulose, EGCG: epigallocatechin gallate, GEL: gelatin, GG: gellan gum, GMS: glycerol monostearate, HPMC: hydroxypropyl methylcellulose, KC: κ-carrageenan, MCT: medium-chain triglycerides, MGs: monoglycerides, PGPR: polyglycerol ricin alkyd ester, RBW: rice bran wax, SA: sodium alginate, SAC: stearic acid, SL: soy lecithin, XG: xanthan gum, WPC: whey protein concentrate, WS: wheat starch.

## Data Availability

Not applicable.
